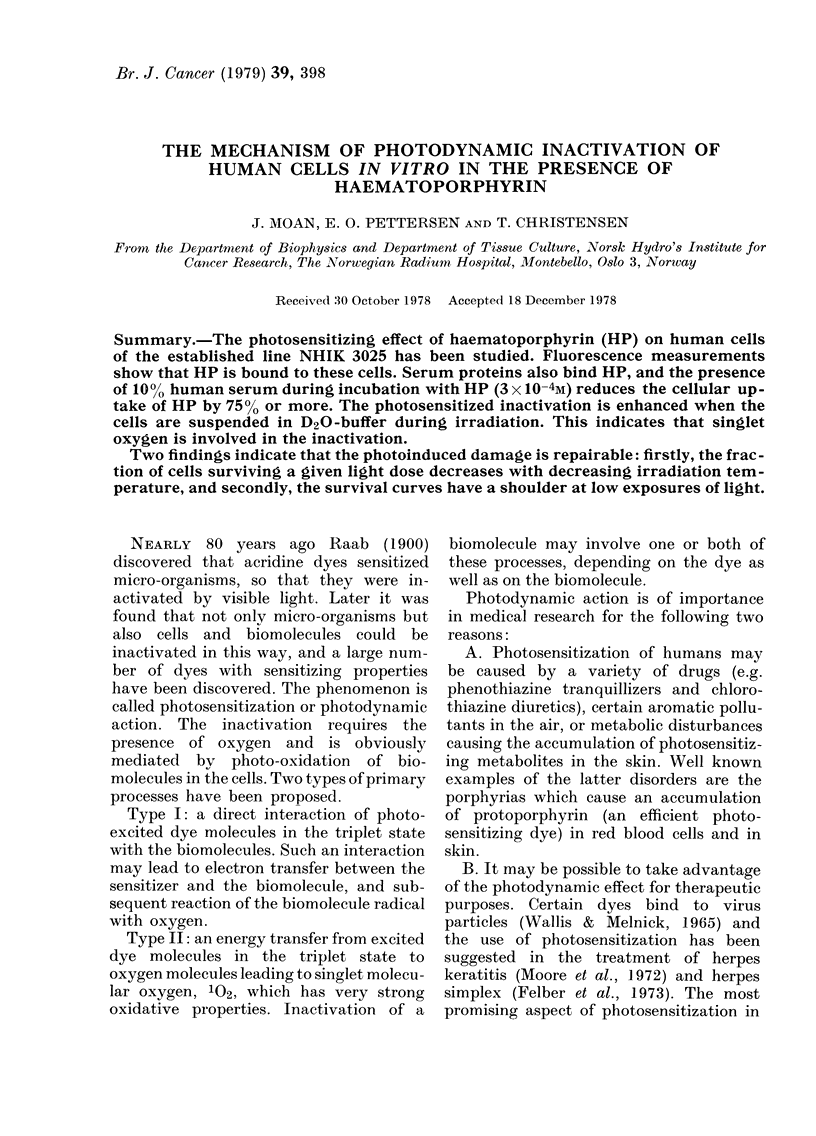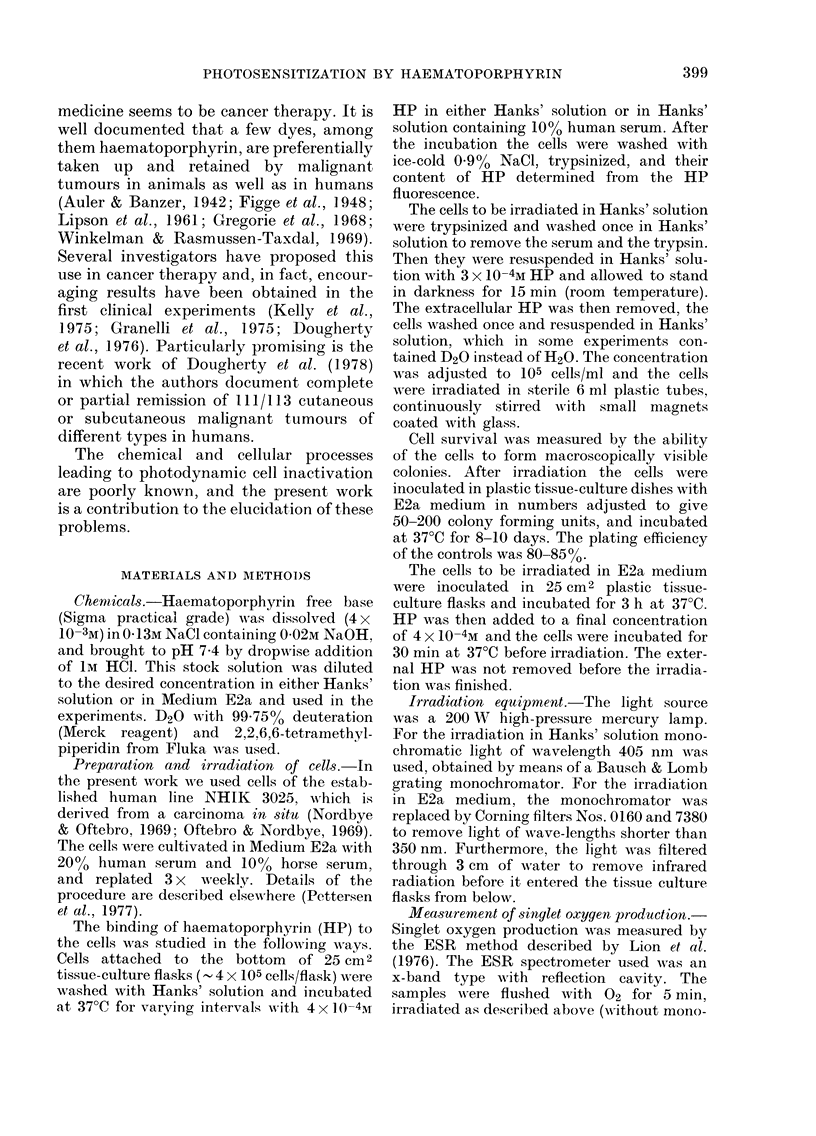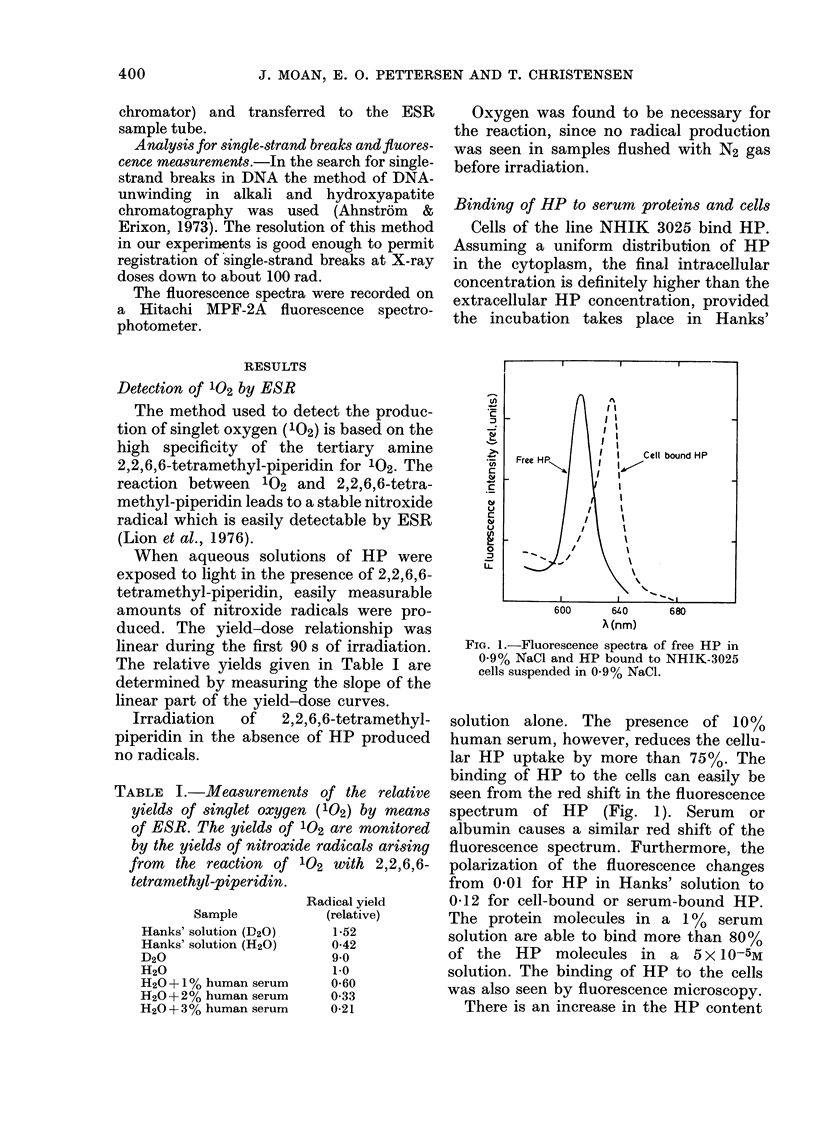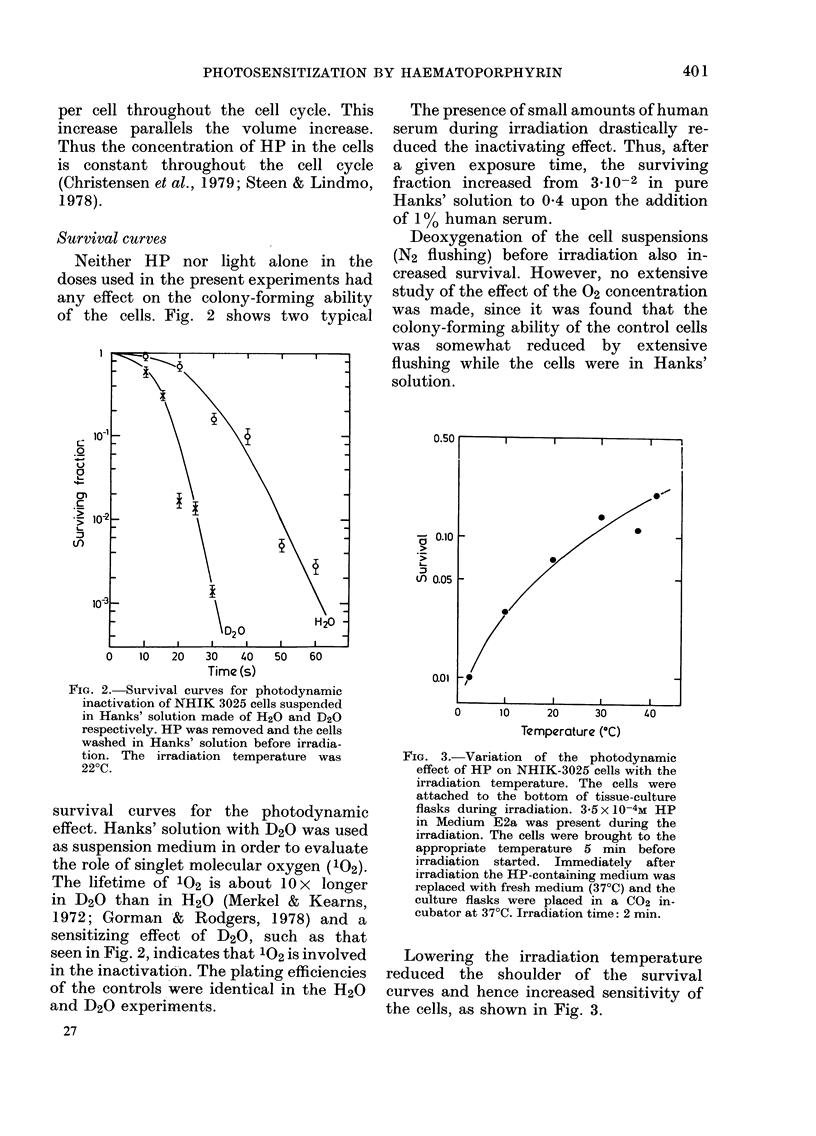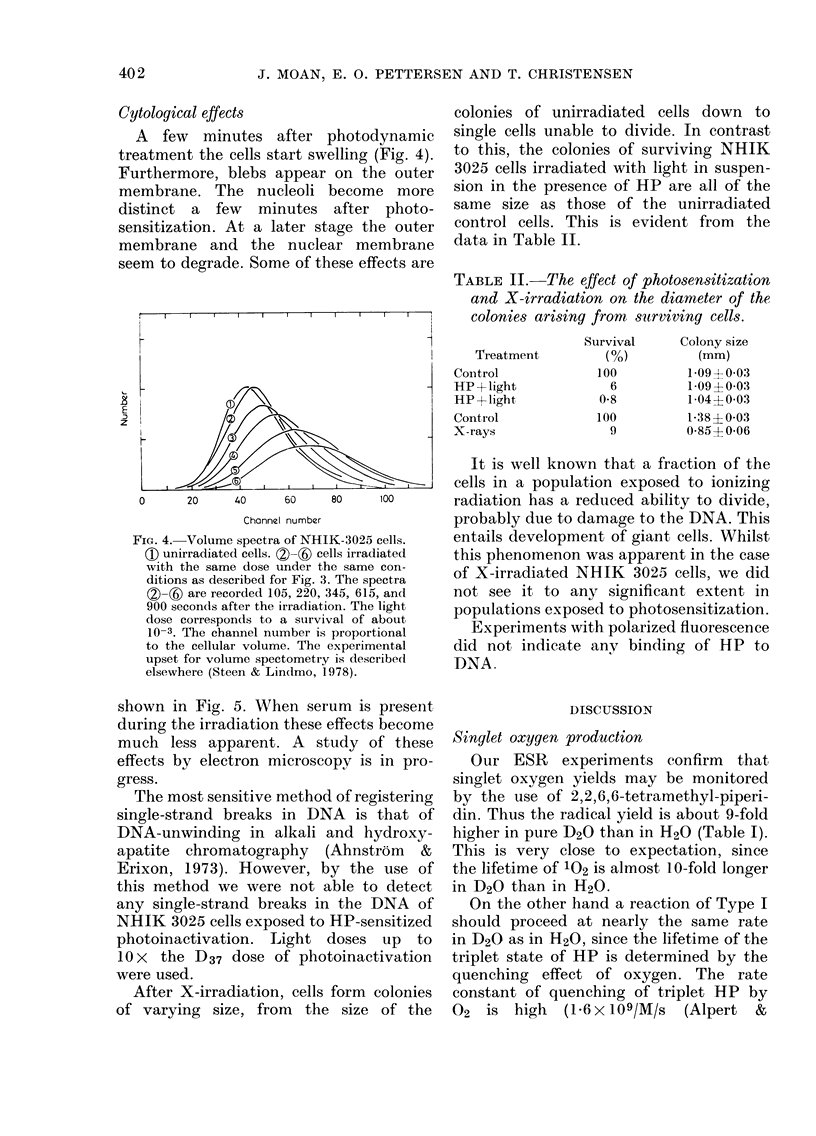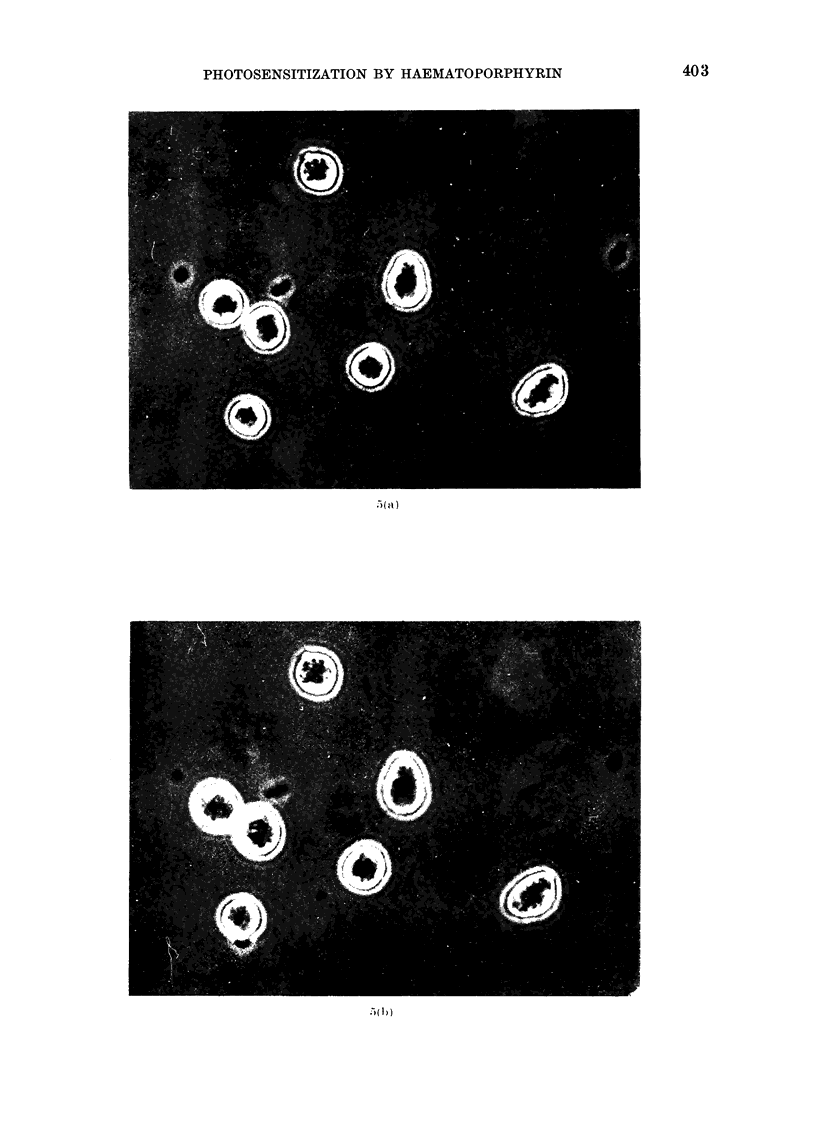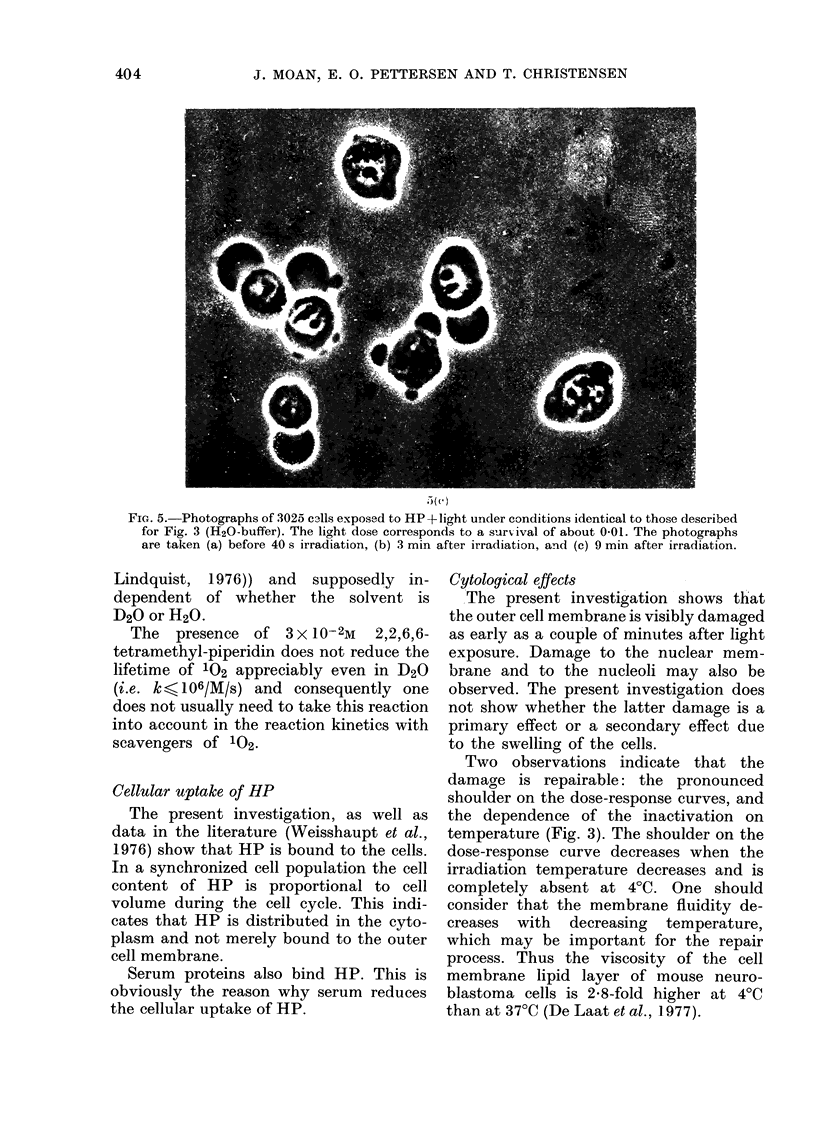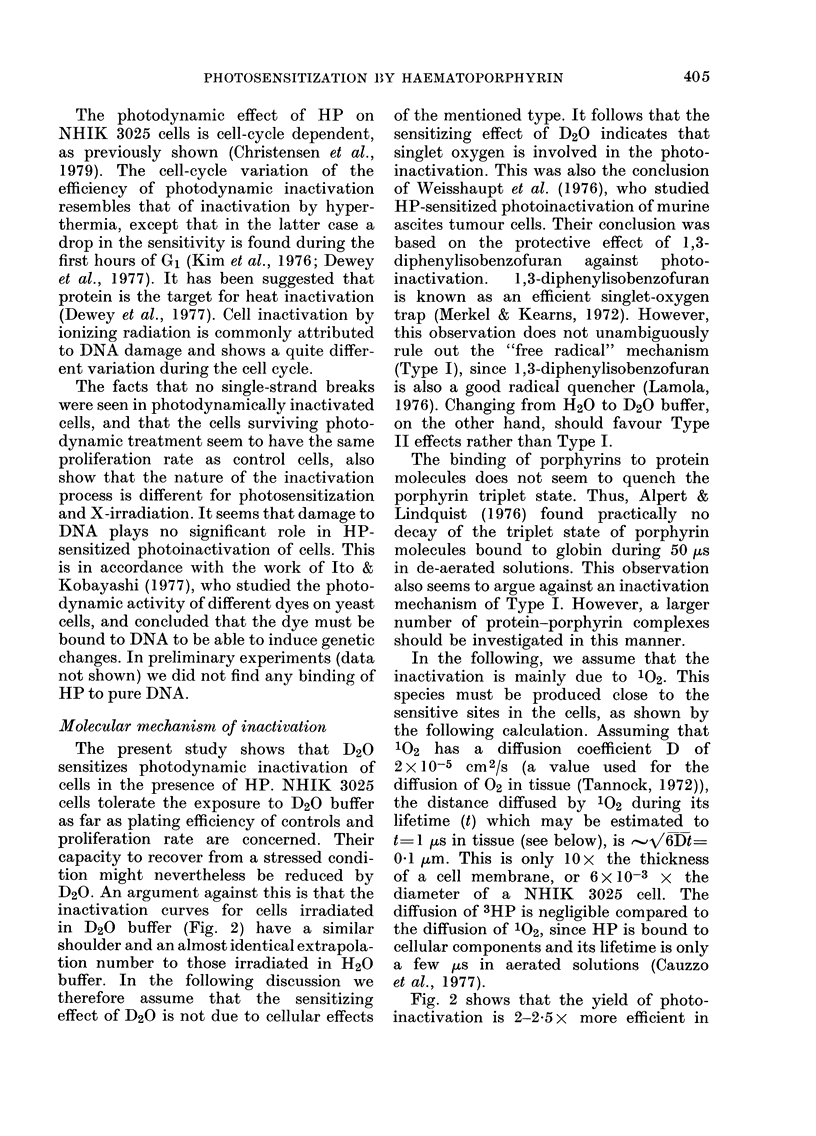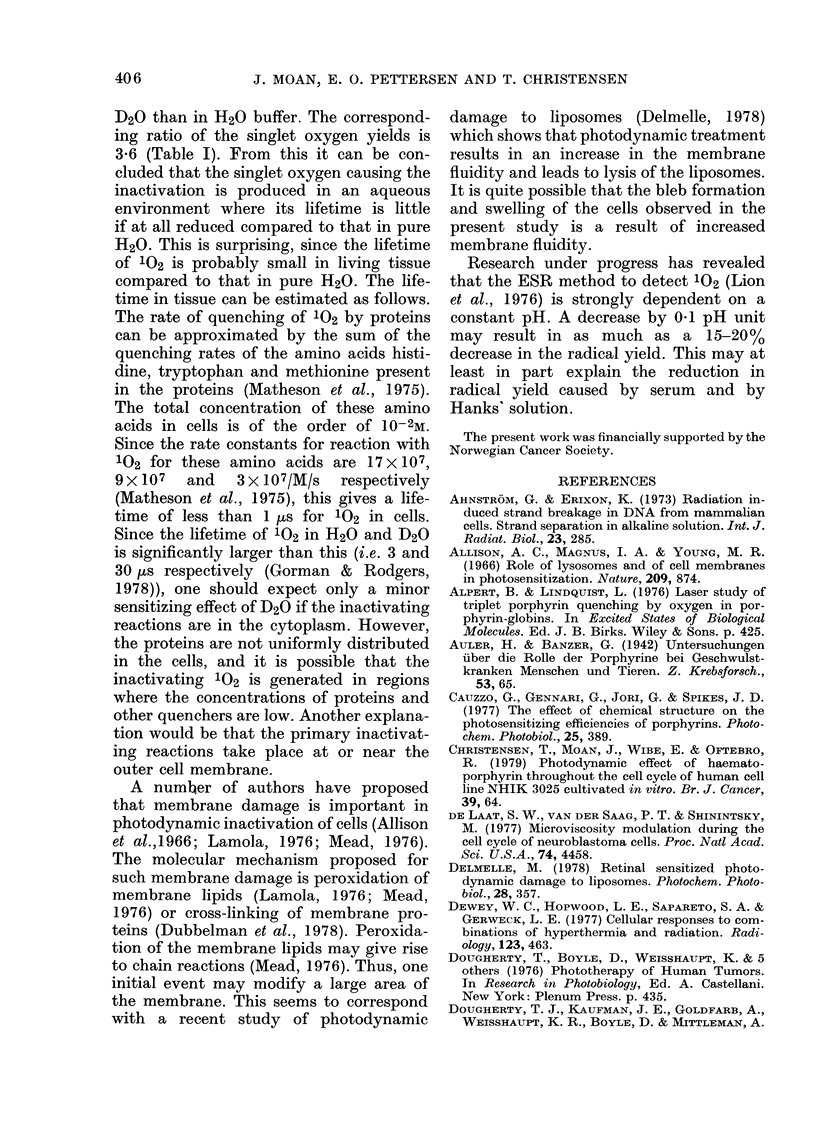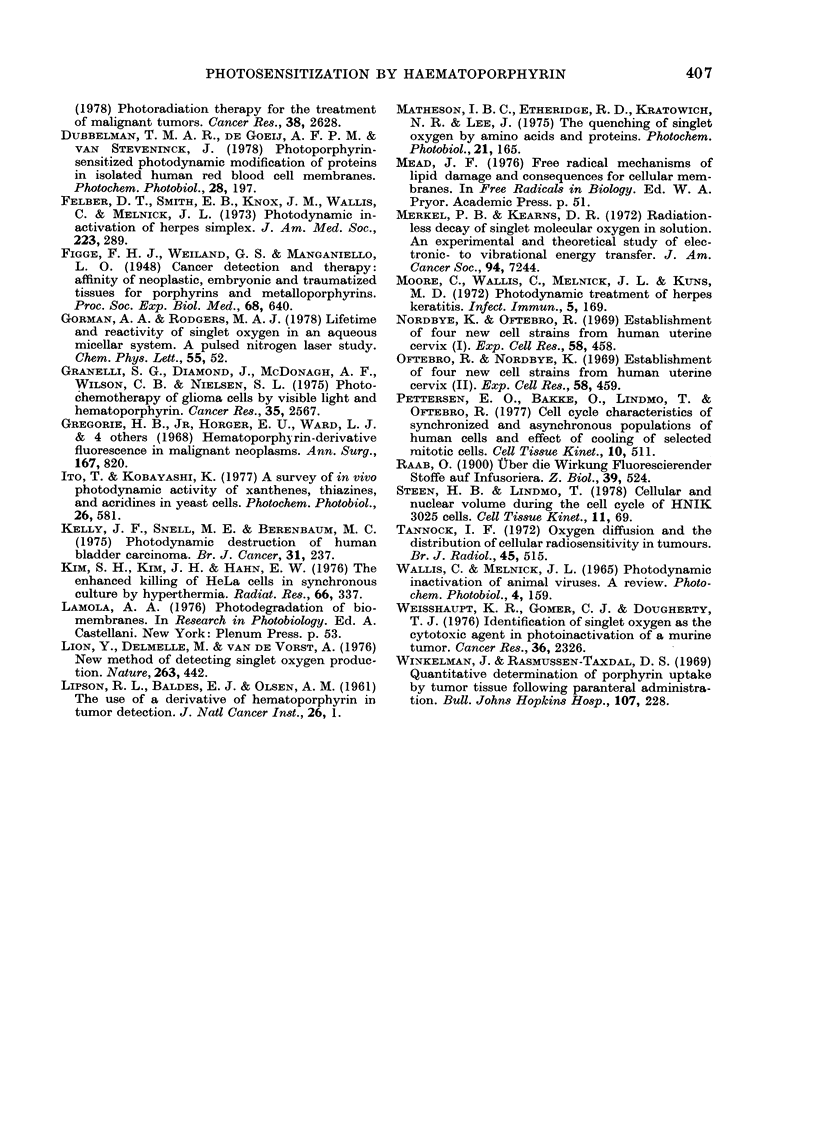# The mechanism of photodynamic inactivation of human cells in vitro in the presence of haematoporphyrin.

**DOI:** 10.1038/bjc.1979.72

**Published:** 1979-04

**Authors:** J. Moan, E. O. Pettersen, T. Christensen

## Abstract

**Images:**


*Br. J. Cancer* (1979) **39**, 398

### THE MECHANISM OF PHOTODYNAMIC INACTIVATION OF HUMAN CELLS *IN VITRO* IN THE PRESENCE OF HAEMATOPORPHYRIN

J. MOAN, E. O. PETTERSEN AND T. CHRISTENSEN

*From the Department of Biophysics and Department of Tissue Culture, Norsk Hydro's Institute for Cancer Research, The Norwegian Radium Hospital, Montebello, Oslo 3, Norway*

Received 30 October 1978 Accepted 18 December 1978

**Summary.—The photosensitizing effect of haematoporphyrin (HP) on human cells of the established line NHIK 3025 has been studied. Fluorescence measurements show that HP is bound to these cells. Serum proteins also bind HP, and the presence of 10% human serum during incubation with HP ($3 \times 10^{-4}$M) reduces the cellular uptake of HP by 75% or more. The photosensitized inactivation is enhanced when the cells are suspended in $D_2O$-buffer during irradiation. This indicates that singlet oxygen is involved in the inactivation.**

**Two findings indicate that the photoinduced damage is repairable: firstly, the fraction of cells surviving a given light dose decreases with decreasing irradiation temperature, and secondly, the survival curves have a shoulder at low exposures of light.**

NEARLY 80 years ago Raab (1900) discovered that acridine dyes sensitized micro-organisms, so that they were inactivated by visible light. Later it was found that not only micro-organisms but also cells and biomolecules could be inactivated in this way, and a large number of dyes with sensitizing properties have been discovered. The phenomenon is called photosensitization or photodynamic action. The inactivation requires the presence of oxygen and is obviously mediated by photo-oxidation of biomolecules in the cells. Two types of primary processes have been proposed.

Type I: a direct interaction of photoexcited dye molecules in the triplet state with the biomolecules. Such an interaction may lead to electron transfer between the sensitizer and the biomolecule, and subsequent reaction of the biomolecule radical with oxygen.

Type II: an energy transfer from excited dye molecules in the triplet state to oxygen molecules leading to singlet molecular oxygen, $^1O_2$, which has very strong oxidative properties. Inactivation of a biomolecule may involve one or both of these processes, depending on the dye as well as on the biomolecule.

Photodynamic action is of importance in medical research for the following two reasons:

A. Photosensitization of humans may be caused by a variety of drugs (e.g. phenothiazine tranquillizers and chlorothiazine diuretics), certain aromatic pollutants in the air, or metabolic disturbances causing the accumulation of photosensitizing metabolites in the skin. Well known examples of the latter disorders are the porphyrias which cause an accumulation of protoporphyrin (an efficient photosensitizing dye) in red blood cells and in skin.

B. It may be possible to take advantage of the photodynamic effect for therapeutic purposes. Certain dyes bind to virus particles (Wallis & Melnick, 1965) and the use of photosensitization has been suggested in the treatment of herpes keratitis (Moore *et al.*, 1972) and herpes simplex (Felber *et al.*, 1973). The most promising aspect of photosensitization in medicine seems to be cancer therapy. It is well documented that a few dyes, among them haematoporphyrin, are preferentially taken up and retained by malignant tumours in animals as well as in humans (Auler & Banzer, 1942; Figge *et al.*, 1948; Lipson *et al.*, 1961; Gregorie *et al.*, 1968; Winkelman & Rasmussen-Taxdal, 1969). Several investigators have proposed this use in cancer therapy and, in fact, encouraging results have been obtained in the first clinical experiments (Kelly *et al.*, 1975; Granelli *et al.*, 1975; Dougherty *et al.*, 1976). Particularly promising is the recent work of Dougherty *et al.* (1978) in which the authors document complete or partial remission of 111/113 cutaneous or subcutaneous malignant tumours of different types in humans.

The chemical and cellular processes leading to photodynamic cell inactivation are poorly known, and the present work is a contribution to the elucidation of these problems.

### MATERIALS AND METHODS

*Chemicals.*—Haematoporphyrin free base (Sigma practical grade) was dissolved ($4 \times 10^{-3}$M) in 0·13M NaCl containing 0·02M NaOH, and brought to pH 7·4 by dropwise addition of 1M HCl. This stock solution was diluted to the desired concentration in either Hanks' solution or in Medium E2a and used in the experiments. $D_2O$ with 99·75% deuteration (Merck reagent) and 2,2,6,6-tetramethylpiperidin from Fluka was used.

*Preparation and irradiation of cells.*—In the present work we used cells of the established human line NHIK 3025, which is derived from a carcinoma *in situ* (Nordbye & Oftebro, 1969; Oftebro & Nordbye, 1969). The cells were cultivated in Medium E2a with 20% human serum and 10% horse serum, and replated 3× weekly. Details of the procedure are described elsewhere (Pettersen *et al.*, 1977).

The binding of haematoporphyrin (HP) to the cells was studied in the following ways. Cells attached to the bottom of 25 cm² tissue-culture flasks ($\sim 4 \times 10^5$ cells/flask) were washed with Hanks' solution and incubated at 37°C for varying intervals with $4 \times 10^{-4}$M HP in either Hanks' solution or in Hanks' solution containing 10% human serum. After the incubation the cells were washed with ice-cold 0·9% NaCl, trypsinized, and their content of HP determined from the HP fluorescence.

The cells to be irradiated in Hanks' solution were trypsinized and washed once in Hanks' solution to remove the serum and the trypsin. Then they were resuspended in Hanks' solution with $3 \times 10^{-4}$M HP and allowed to stand in darkness for 15 min (room temperature). The extracellular HP was then removed, the cells washed once and resuspended in Hanks' solution, which in some experiments contained $D_2O$ instead of $H_2O$. The concentration was adjusted to $10^5$ cells/ml and the cells were irradiated in sterile 6 ml plastic tubes, continuously stirred with small magnets coated with glass.

Cell survival was measured by the ability of the cells to form macroscopically visible colonies. After irradiation the cells were inoculated in plastic tissue-culture dishes with E2a medium in numbers adjusted to give 50–200 colony forming units, and incubated at 37°C for 8–10 days. The plating efficiency of the controls was 80–85%.

The cells to be irradiated in E2a medium were inoculated in 25 cm² plastic tissue-culture flasks and incubated for 3 h at 37°C. HP was then added to a final concentration of $4 \times 10^{-4}$M and the cells were incubated for 30 min at 37°C before irradiation. The external HP was not removed before the irradiation was finished.

*Irradiation equipment.*—The light source was a 200 W high-pressure mercury lamp. For the irradiation in Hanks' solution monochromatic light of wavelength 405 nm was used, obtained by means of a Bausch & Lomb grating monochromator. For the irradiation in E2a medium, the monochromator was replaced by Corning filters Nos. 0160 and 7380 to remove light of wave-lengths shorter than 350 nm. Furthermore, the light was filtered through 3 cm of water to remove infrared radiation before it entered the tissue culture flasks from below.

*Measurement of singlet oxygen production.*—Singlet oxygen production was measured by the ESR method described by Lion *et al.* (1976). The ESR spectrometer used was an x-band type with reflection cavity. The samples were flushed with $O_2$ for 5 min, irradiated as described above (without monochromator) and transferred to the ESR sample tube.

*Analysis for single-strand breaks and fluorescence measurements.*—In the search for single-strand breaks in DNA the method of DNA-unwinding in alkali and hydroxyapatite chromatography was used (Ahnström & Erixon, 1973). The resolution of this method in our experiments is good enough to permit registration of single-strand breaks at X-ray doses down to about 100 rad.

The fluorescence spectra were recorded on a Hitachi MPF-2A fluorescence spectrophotometer.

### RESULTS

### *Detection of* $^1O_2$ *by ESR*

The method used to detect the production of singlet oxygen ($^1O_2$) is based on the high specificity of the tertiary amine 2,2,6,6-tetramethyl-piperidin for $^1O_2$. The reaction between $^1O_2$ and 2,2,6,6-tetramethyl-piperidin leads to a stable nitroxide radical which is easily detectable by ESR (Lion *et al.*, 1976).

When aqueous solutions of HP were exposed to light in the presence of 2,2,6,6-tetramethyl-piperidin, easily measurable amounts of nitroxide radicals were produced. The yield–dose relationship was linear during the first 90 s of irradiation. The relative yields given in Table I are determined by measuring the slope of the linear part of the yield–dose curves.

Irradiation of 2,2,6,6-tetramethyl-piperidin in the absence of HP produced no radicals.

TABLE I.—*Measurements of the relative yields of singlet oxygen* ($^1O_2$) *by means of ESR. The yields of* $^1O_2$ *are monitored by the yields of nitroxide radicals arising from the reaction of* $^1O_2$ *with* 2,2,6,6-*tetramethyl-piperidin.*

| Sample | Radical yield (relative) |
|---|---|
| Hanks' solution ($D_2O$) | 1·52 |
| Hanks' solution ($H_2O$) | 0·42 |
| $D_2O$ | 9·0 |
| $H_2O$ | 1·0 |
| $H_2O$ + 1% human serum | 0·60 |
| $H_2O$ + 2% human serum | 0·33 |
| $H_2O$ + 3% human serum | 0·21 |

Oxygen was found to be necessary for the reaction, since no radical production was seen in samples flushed with $N_2$ gas before irradiation.

### *Binding of HP to serum proteins and cells*

Cells of the line NHIK 3025 bind HP. Assuming a uniform distribution of HP in the cytoplasm, the final intracellular concentration is definitely higher than the extracellular HP concentration, provided the incubation takes place in Hanks'

FIG. 1.—Fluorescence spectra of free HP in 0·9% NaCl and HP bound to NHIK-3025 cells suspended in 0·9% NaCl.

solution alone. The presence of 10% human serum, however, reduces the cellular HP uptake by more than 75%. The binding of HP to the cells can easily be seen from the red shift in the fluorescence spectrum of HP (Fig. 1). Serum or albumin causes a similar red shift of the fluorescence spectrum. Furthermore, the polarization of the fluorescence changes from 0·01 for HP in Hanks' solution to 0·12 for cell-bound or serum-bound HP. The protein molecules in a 1% serum solution are able to bind more than 80% of the HP molecules in a $5 \times 10^{-5}$M solution. The binding of HP to the cells was also seen by fluorescence microscopy.

There is an increase in the HP content per cell throughout the cell cycle. This increase parallels the volume increase. Thus the concentration of HP in the cells is constant throughout the cell cycle (Christensen *et al.*, 1979; Steen & Lindmo, 1978).

*Survival curves*

Neither HP nor light alone in the doses used in the present experiments had any effect on the colony-forming ability of the cells. Fig. 2 shows two typical

Fig. 2.—Survival curves for photodynamic inactivation of NHIK 3025 cells suspended in Hanks' solution made of $H_2O$ and $D_2O$ respectively. HP was removed and the cells washed in Hanks' solution before irradiation. The irradiation temperature was 22°C.

survival curves for the photodynamic effect. Hanks' solution with $D_2O$ was used as suspension medium in order to evaluate the role of singlet molecular oxygen ($^1O_2$). The lifetime of $^1O_2$ is about 10× longer in $D_2O$ than in $H_2O$ (Merkel & Kearns, 1972; Gorman & Rodgers, 1978) and a sensitizing effect of $D_2O$, such as that seen in Fig. 2, indicates that $^1O_2$ is involved in the inactivation. The plating efficiencies of the controls were identical in the $H_2O$ and $D_2O$ experiments.

The presence of small amounts of human serum during irradiation drastically reduced the inactivating effect. Thus, after a given exposure time, the surviving fraction increased from $3{\cdot}10^{-2}$ in pure Hanks' solution to 0·4 upon the addition of 1% human serum.

Deoxygenation of the cell suspensions ($N_2$ flushing) before irradiation also increased survival. However, no extensive study of the effect of the $O_2$ concentration was made, since it was found that the colony-forming ability of the control cells was somewhat reduced by extensive flushing while the cells were in Hanks' solution.

Fig. 3.—Variation of the photodynamic effect of HP on NHIK-3025 cells with the irradiation temperature. The cells were attached to the bottom of tissue-culture flasks during irradiation. $3{\cdot}5\times10^{-4}$M HP in Medium E2a was present during the irradiation. The cells were brought to the appropriate temperature 5 min before irradiation started. Immediately after irradiation the HP-containing medium was replaced with fresh medium (37°C) and the culture flasks were placed in a $CO_2$ incubator at 37°C. Irradiation time: 2 min.

Lowering the irradiation temperature reduced the shoulder of the survival curves and hence increased sensitivity of the cells, as shown in Fig. 3.

*Cytological effects*

A few minutes after photodynamic treatment the cells start swelling (Fig. 4). Furthermore, blebs appear on the outer membrane. The nucleoli become more distinct a few minutes after photosensitization. At a later stage the outer membrane and the nuclear membrane seem to degrade. Some of these effects are shown in Fig. 5. When serum is present during the irradiation these effects become much less apparent. A study of these effects by electron microscopy is in progress.

FIG. 4.—Volume spectra of NHIK-3025 cells. ① unirradiated cells. ②–⑥ cells irradiated with the same dose under the same conditions as described for Fig. 3. The spectra ②–⑥ are recorded 105, 220, 345, 615, and 900 seconds after the irradiation. The light dose corresponds to a survival of about $10^{-3}$. The channel number is proportional to the cellular volume. The experimental upset for volume spectometry is described elsewhere (Steen & Lindmo, 1978).

The most sensitive method of registering single-strand breaks in DNA is that of DNA-unwinding in alkali and hydroxyapatite chromatography (Ahnström & Erixon, 1973). However, by the use of this method we were not able to detect any single-strand breaks in the DNA of NHIK 3025 cells exposed to HP-sensitized photoinactivation. Light doses up to $10\times$ the $D_{37}$ dose of photoinactivation were used.

After X-irradiation, cells form colonies of varying size, from the size of the colonies of unirradiated cells down to single cells unable to divide. In contrast to this, the colonies of surviving NHIK 3025 cells irradiated with light in suspension in the presence of HP are all of the same size as those of the unirradiated control cells. This is evident from the data in Table II.

TABLE II.—*The effect of photosensitization and X-irradiation on the diameter of the colonies arising from surviving cells.*

| Treatment | Survival (%) | Colony size (mm) |
|---|---|---|
| Control | 100 | $1{\cdot}09 \pm 0{\cdot}03$ |
| HP+light | 6 | $1{\cdot}09 \pm 0{\cdot}03$ |
| HP+light | 0·8 | $1{\cdot}04 \pm 0{\cdot}03$ |
| Control | 100 | $1{\cdot}38 \pm 0{\cdot}03$ |
| X-rays | 9 | $0{\cdot}85 \pm 0{\cdot}06$ |

It is well known that a fraction of the cells in a population exposed to ionizing radiation has a reduced ability to divide, probably due to damage to the DNA. This entails development of giant cells. Whilst this phenomenon was apparent in the case of X-irradiated NHIK 3025 cells, we did not see it to any significant extent in populations exposed to photosensitization.

Experiments with polarized fluorescence did not indicate any binding of HP to DNA.

### DISCUSSION

*Singlet oxygen production*

Our ESR experiments confirm that singlet oxygen yields may be monitored by the use of 2,2,6,6-tetramethyl-piperidin. Thus the radical yield is about 9-fold higher in pure $D_2O$ than in $H_2O$ (Table I). This is very close to expectation, since the lifetime of $^1O_2$ is almost 10-fold longer in $D_2O$ than in $H_2O$.

On the other hand a reaction of Type I should proceed at nearly the same rate in $D_2O$ as in $H_2O$, since the lifetime of the triplet state of HP is determined by the quenching effect of oxygen. The rate constant of quenching of triplet HP by $O_2$ is high ($1{\cdot}6\times10^9$/M/s (Alpert &

5(a)

5(b)

5(c)

FIG. 5.—Photographs of 3025 cells exposed to HP + light under conditions identical to those described for Fig. 3 ($H_2O$-buffer). The light dose corresponds to a survival of about 0·01. The photographs are taken (a) before 40 s irradiation, (b) 3 min after irradiation, and (c) 9 min after irradiation.

Lindquist, 1976)) and supposedly independent of whether the solvent is $D_2O$ or $H_2O$.

The presence of $3 \times 10^{-2}$M 2,2,6,6-tetramethyl-piperidin does not reduce the lifetime of $^1O_2$ appreciably even in $D_2O$ (*i.e.* $k \leqslant 10^6$/M/s) and consequently one does not usually need to take this reaction into account in the reaction kinetics with scavengers of $^1O_2$.

*Cellular uptake of HP*

The present investigation, as well as data in the literature (Weisshaupt *et al.*, 1976) show that HP is bound to the cells. In a synchronized cell population the cell content of HP is proportional to cell volume during the cell cycle. This indicates that HP is distributed in the cytoplasm and not merely bound to the outer cell membrane.

Serum proteins also bind HP. This is obviously the reason why serum reduces the cellular uptake of HP.

*Cytological effects*

The present investigation shows that the outer cell membrane is visibly damaged as early as a couple of minutes after light exposure. Damage to the nuclear membrane and to the nucleoli may also be observed. The present investigation does not show whether the latter damage is a primary effect or a secondary effect due to the swelling of the cells.

Two observations indicate that the damage is repairable: the pronounced shoulder on the dose-response curves, and the dependence of the inactivation on temperature (Fig. 3). The shoulder on the dose-response curve decreases when the irradiation temperature decreases and is completely absent at 4°C. One should consider that the membrane fluidity decreases with decreasing temperature, which may be important for the repair process. Thus the viscosity of the cell membrane lipid layer of mouse neuroblastoma cells is 2·8-fold higher at 4°C than at 37°C (De Laat *et al.*, 1977).

The photodynamic effect of HP on NHIK 3025 cells is cell-cycle dependent, as previously shown (Christensen *et al.*, 1979). The cell-cycle variation of the efficiency of photodynamic inactivation resembles that of inactivation by hyperthermia, except that in the latter case a drop in the sensitivity is found during the first hours of $G_1$ (Kim *et al.*, 1976; Dewey *et al.*, 1977). It has been suggested that protein is the target for heat inactivation (Dewey *et al.*, 1977). Cell inactivation by ionizing radiation is commonly attributed to DNA damage and shows a quite different variation during the cell cycle.

The facts that no single-strand breaks were seen in photodynamically inactivated cells, and that the cells surviving photodynamic treatment seem to have the same proliferation rate as control cells, also show that the nature of the inactivation process is different for photosensitization and X-irradiation. It seems that damage to DNA plays no significant role in HP-sensitized photoinactivation of cells. This is in accordance with the work of Ito & Kobayashi (1977), who studied the photodynamic activity of different dyes on yeast cells, and concluded that the dye must be bound to DNA to be able to induce genetic changes. In preliminary experiments (data not shown) we did not find any binding of HP to pure DNA.

*Molecular mechanism of inactivation*

The present study shows that $D_2O$ sensitizes photodynamic inactivation of cells in the presence of HP. NHIK 3025 cells tolerate the exposure to $D_2O$ buffer as far as plating efficiency of controls and proliferation rate are concerned. Their capacity to recover from a stressed condition might nevertheless be reduced by $D_2O$. An argument against this is that the inactivation curves for cells irradiated in $D_2O$ buffer (Fig. 2) have a similar shoulder and an almost identical extrapolation number to those irradiated in $H_2O$ buffer. In the following discussion we therefore assume that the sensitizing effect of $D_2O$ is not due to cellular effects of the mentioned type. It follows that the sensitizing effect of $D_2O$ indicates that singlet oxygen is involved in the photoinactivation. This was also the conclusion of Weisshaupt *et al.* (1976), who studied HP-sensitized photoinactivation of murine ascites tumour cells. Their conclusion was based on the protective effect of 1,3-diphenylisobenzofuran against photoinactivation. 1,3-diphenylisobenzofuran is known as an efficient singlet-oxygen trap (Merkel & Kearns, 1972). However, this observation does not unambiguously rule out the "free radical" mechanism (Type I), since 1,3-diphenylisobenzofuran is also a good radical quencher (Lamola, 1976). Changing from $H_2O$ to $D_2O$ buffer, on the other hand, should favour Type II effects rather than Type I.

The binding of porphyrins to protein molecules does not seem to quench the porphyrin triplet state. Thus, Alpert & Lindquist (1976) found practically no decay of the triplet state of porphyrin molecules bound to globin during 50 $\mu$s in de-aerated solutions. This observation also seems to argue against an inactivation mechanism of Type I. However, a larger number of protein–porphyrin complexes should be investigated in this manner.

In the following, we assume that the inactivation is mainly due to $^1O_2$. This species must be produced close to the sensitive sites in the cells, as shown by the following calculation. Assuming that $^1O_2$ has a diffusion coefficient D of $2\times10^{-5}$ cm$^2$/s (a value used for the diffusion of $O_2$ in tissue (Tannock, 1972)), the distance diffused by $^1O_2$ during its lifetime ($t$) which may be estimated to $t=1$ $\mu$s in tissue (see below), is $\sim\sqrt{6Dt}=$ 0·1 $\mu$m. This is only 10$\times$ the thickness of a cell membrane, or $6\times10^{-3}$ $\times$ the diameter of a NHIK 3025 cell. The diffusion of $^3$HP is negligible compared to the diffusion of $^1O_2$, since HP is bound to cellular components and its lifetime is only a few $\mu$s in aerated solutions (Cauzzo *et al.*, 1977).

Fig. 2 shows that the yield of photoinactivation is 2–2·5$\times$ more efficient in

$D_2O$ than in $H_2O$ buffer. The corresponding ratio of the singlet oxygen yields is 3·6 (Table I). From this it can be concluded that the singlet oxygen causing the inactivation is produced in an aqueous environment where its lifetime is little if at all reduced compared to that in pure $H_2O$. This is surprising, since the lifetime of $^1O_2$ is probably small in living tissue compared to that in pure $H_2O$. The lifetime in tissue can be estimated as follows. The rate of quenching of $^1O_2$ by proteins can be approximated by the sum of the quenching rates of the amino acids histidine, tryptophan and methionine present in the proteins (Matheson *et al.*, 1975). The total concentration of these amino acids in cells is of the order of $10^{-2}$M. Since the rate constants for reaction with $^1O_2$ for these amino acids are $17\times10^7$, $9\times10^7$ and $3\times10^7$/M/s respectively (Matheson *et al.*, 1975), this gives a lifetime of less than 1 $\mu$s for $^1O_2$ in cells. Since the lifetime of $^1O_2$ in $H_2O$ and $D_2O$ is significantly larger than this (*i.e.* 3 and 30 $\mu$s respectively (Gorman & Rodgers, 1978)), one should expect only a minor sensitizing effect of $D_2O$ if the inactivating reactions are in the cytoplasm. However, the proteins are not uniformly distributed in the cells, and it is possible that the inactivating $^1O_2$ is generated in regions where the concentrations of proteins and other quenchers are low. Another explanation would be that the primary inactivating reactions take place at or near the outer cell membrane.

A number of authors have proposed that membrane damage is important in photodynamic inactivation of cells (Allison *et al.*,1966; Lamola, 1976; Mead, 1976). The molecular mechanism proposed for such membrane damage is peroxidation of membrane lipids (Lamola, 1976; Mead, 1976) or cross-linking of membrane proteins (Dubbelman *et al.*, 1978). Peroxidation of the membrane lipids may give rise to chain reactions (Mead, 1976). Thus, one initial event may modify a large area of the membrane. This seems to correspond with a recent study of photodynamic damage to liposomes (Delmelle, 1978) which shows that photodynamic treatment results in an increase in the membrane fluidity and leads to lysis of the liposomes. It is quite possible that the bleb formation and swelling of the cells observed in the present study is a result of increased membrane fluidity.

Research under progress has revealed that the ESR method to detect $^1O_2$ (Lion *et al.*, 1976) is strongly dependent on a constant pH. A decrease by 0·1 pH unit may result in as much as a 15–20% decrease in the radical yield. This may at least in part explain the reduction in radical yield caused by serum and by Hanks' solution.

The present work was financially supported by the Norwegian Cancer Society.

### REFERENCES

Ahnström, G. & Erixon, K. (1973) Radiation induced strand breakage in DNA from mammalian cells. Strand separation in alkaline solution. *Int. J. Radiat. Biol.*, **23**, 285.

Allison, A. C., Magnus, I. A. & Young, M. R. (1966) Role of lysosomes and of cell membranes in photosensitization. *Nature*, **209**, 874.

Alpert, B. & Lindquist, L. (1976) Laser study of triplet porphyrin quenching by oxygen in porphyrin-globins. In *Excited States of Biological Molecules*. Ed. J. B. Birks. Wiley & Sons. p. 425.

Auler, H. & Banzer, G. (1942) Untersuchungen über die Rolle der Porphyrine bei Geschwulstkranken Menschen und Tieren. *Z. Krebsforsch.*, **53**, 65.

Cauzzo, G., Gennari, G., Jori, G. & Spikes, J. D. (1977) The effect of chemical structure on the photosensitizing efficiencies of porphyrins. *Photochem. Photobiol.*, **25**, 389.

Christensen, T., Moan, J., Wibe, E. & Oftebro, R. (1979) Photodynamic effect of haematoporphyrin throughout the cell cycle of human cell line NHIK 3025 cultivated *in vitro*. *Br. J. Cancer*, **39**, 64.

de Laat, S. W., van der Saag, P. T. & Shinintsky, M. (1977) Microviscosity modulation during the cell cycle of neuroblastoma cells. *Proc. Natl Acad. Sci. U.S.A.*, **74**, 4458.

Delmelle, M. (1978) Retinal sensitized photodynamic damage to liposomes. *Photochem. Photobiol.*, **28**, 357.

Dewey, W. C., Hopwood, L. E., Sapareto, S. A. & Gerweck, L. E. (1977) Cellular responses to combinations of hyperthermia and radiation. *Radiology*, **123**, 463.

Dougherty, T., Boyle, D., Weisshaupt, K. & 5 others (1976) Phototherapy of Human Tumors. In *Research in Photobiology*, Ed. A. Castellani. New York: Plenum Press. p. 435.

Dougherty, T. J., Kaufman, J. E., Goldfarb, A., Weisshaupt, K. R., Boyle, D. & Mittleman, A.

(1978) Photoradiation therapy for the treatment of malignant tumors. *Cancer Res.*, **38**, 2628.

DUBBELMAN, T. M. A. R., DE GOEIJ, A. F. P. M. & VAN STEVENINCK, J. (1978) Photoporphyrin-sensitized photodynamic modification of proteins in isolated human red blood cell membranes. *Photochem. Photobiol.*, **28**, 197.

FELBER, D. T., SMITH, E. B., KNOX, J. M., WALLIS, C. & MELNICK, J. L. (1973) Photodynamic inactivation of herpes simplex. *J. Am. Med. Soc.*, **223**, 289.

FIGGE, F. H. J., WEILAND, G. S. & MANGANIELLO, L. O. (1948) Cancer detection and therapy: affinity of neoplastic, embryonic and traumatized tissues for porphyrins and metalloporphyrins. *Proc. Soc. Exp. Biol. Med.*, **68**, 640.

GORMAN, A. A. & RODGERS, M. A. J. (1978) Lifetime and reactivity of singlet oxygen in an aqueous micellar system. A pulsed nitrogen laser study. *Chem. Phys. Lett.*, **55**, 52.

GRANELLI, S. G., DIAMOND, J., MCDONAGH, A. F., WILSON, C. B. & NIELSEN, S. L. (1975) Photochemotherapy of glioma cells by visible light and hematoporphyrin. *Cancer Res.*, **35**, 2567.

GREGORIE, H. B., JR, HORGER, E. U., WARD, L. J. & 4 others (1968) Hematoporphyrin-derivative fluorescence in malignant neoplasms. *Ann. Surg.*, **167**, 820.

ITO, T. & KOBAYASHI, K. (1977) A survey of *in vivo* photodynamic activity of xanthenes, thiazines, and acridines in yeast cells. *Photochem. Photobiol.*, **26**, 581.

KELLY, J. F., SNELL, M. E. & BERENBAUM, M. C. (1975) Photodynamic destruction of human bladder carcinoma. *Br. J. Cancer*, **31**, 237.

KIM, S. H., KIM, J. H. & HAHN, E. W. (1976) The enhanced killing of HeLa cells in synchronous culture by hyperthermia. *Radiat. Res.*, **66**, 337.

LAMOLA, A. A. (1976) Photodegradation of biomembranes. In *Research in Photobiology*. Ed. A. Castellani. New York: Plenum Press. p. 53.

LION, Y., DELMELLE, M. & VAN DE VORST, A. (1976) New method of detecting singlet oxygen production. *Nature*, **263**, 442.

LIPSON, R. L., BALDES, E. J. & OLSEN, A. M. (1961) The use of a derivative of hematoporphyrin in tumor detection. *J. Natl Cancer Inst.*, **26**, 1.

MATHESON, I. B. C., ETHERIDGE, R. D., KRATOWICH, N. R. & LEE, J. (1975) The quenching of singlet oxygen by amino acids and proteins. *Photochem. Photobiol.*, **21**, 165.

MEAD, J. F. (1976) Free radical mechanisms of lipid damage and consequences for cellular membranes. In *Free Radicals in Biology*. Ed. W. A. Pryor. Academic Press. p. 51.

MERKEL, P. B. & KEARNS, D. R. (1972) Radiationless decay of singlet molecular oxygen in solution. An experimental and theoretical study of electronic- to vibrational energy transfer. *J. Am. Cancer Soc.*, **94**, 7244.

MOORE, C., WALLIS, C., MELNICK, J. L. & KUNS, M. D. (1972) Photodynamic treatment of herpes keratitis. *Infect. Immun.*, **5**, 169.

NORDBYE, K. & OFTEBRO, R. (1969) Establishment of four new cell strains from human uterine cervix (I). *Exp. Cell Res.*, **58**, 458.

OFTEBRO, R. & NORDBYE, K. (1969) Establishment of four new cell strains from human uterine cervix (II). *Exp. Cell Res.*, **58**, 459.

PETTERSEN, E. O., BAKKE, O., LINDMO, T. & OFTEBRO, R. (1977) Cell cycle characteristics of synchronized and asynchronous populations of human cells and effect of cooling of selected mitotic cells. *Cell Tissue Kinet.*, **10**, 511.

RAAB, O. (1900) Über die Wirkung Fluorescierender Stoffe auf Infusoriera. *Z. Biol.*, **39**, 524.

STEEN, H. B. & LINDMO, T. (1978) Cellular and nuclear volume during the cell cycle of HNIK 3025 cells. *Cell Tissue Kinet.*, **11**, 69.

TANNOCK, I. F. (1972) Oxygen diffusion and the distribution of cellular radiosensitivity in tumours. *Br. J. Radiol.*, **45**, 515.

WALLIS, C. & MELNICK, J. L. (1965) Photodynamic inactivation of animal viruses. A review. *Photochem. Photobiol.*, **4**, 159.

WEISSHAUPT, K. R., GOMER, C. J. & DOUGHERTY, T. J. (1976) Identification of singlet oxygen as the cytotoxic agent in photoinactivation of a murine tumor. *Cancer Res.*, **36**, 2326.

WINKELMAN, J. & RASMUSSEN-TAXDAL, D. S. (1969) Quantitative determination of porphyrin uptake by tumor tissue following paranteral administration. *Bull. Johns Hopkins Hosp.*, **107**, 228.